# A prognostic model incorporating MCP-1 and IL-8 for early risk stratification in acute ischemic stroke patients receiving intravenous thrombolysis: a retrospective development with temporal validation

**DOI:** 10.3389/fneur.2026.1765374

**Published:** 2026-06-04

**Authors:** Huiyuan Zhang, Lei Si, Yamin Song, Huiqian Guo, Fengyuan Jiao, Jialiang Liu, Yu Hao

**Affiliations:** 1 Department of Neurology, Liaocheng People’s Hospital, Liaocheng, Shandong, China; 2 Department of Clinical Laboratory, Liaocheng People’s Hospital, Liaocheng, Shandong, China; 3 Nanchang University Queen Mary College, Nanchang, Jiangxi, China

**Keywords:** acute ischemic stroke, Interleukin-8, intravenous thrombolysis, monocyte chemoattractant protein-1, prognostic model

## Abstract

**Objective:**

To investigate the expression characteristics of monocyte chemoattractant protein-1 (MCP-1) and interleukin-8 (IL-8) in patients with acute ischemic stroke (AIS) who received intravenous thrombolysis and their relationship with clinical prognosis.

**Methods:**

This retrospective study included 300 patients with AIS who received intravenous thrombolysis. Patients were chronologically divided into a development cohort (*n* = 200) and a temporal validation cohort (*n* = 100). A dynamic substudy cohort (*n* = 150) underwent serial sampling at 24 and 72 h. Patients were stratified by 90-day mRS score into a good outcome group (mRS ≤ 2, *n* = 192) and a poor outcome group (mRS > 2, *n* = 108). A separate healthy control group (*n* = 100) was included for reference. Multivariate logistic regression was performed using only variables available at admission (age, NIHSS score, infarct volume, MCP-1, IL-8) to build a prognostic nomogram for early risk stratification. Internal bootstrap validation and temporal validation were conducted, and decision curve analysis (DCA) assessed the net clinical benefit of adding biomarkers to a clinical baseline model.

**Results:**

Within the AIS cohort, MCP-1 and IL-8 levels were significantly higher in the poor outcome group compared with the good outcome group (both *p* < 0.001). Dynamic analysis revealed sustained elevation at 72 h only in the poor outcome group (interaction *p* < 0.001). Multivariate analysis in the development cohort identified age, NIHSS, infarct volume, MCP-1, and IL-8 as independent predictors. The nomogram showed a C-index of 0.872 in the development cohort and 0.842 in the temporal validation cohort, with good calibration. Decision curve analysis confirmed net clinical benefit in both cohorts. The AUC for predicting poor prognosis was 0.773 for MCP-1, 0.742 for IL-8, and 0.839 for the combination of both markers. Decision curve analysis confirmed the added clinical net benefit of the biomarker-enhanced model.

**Conclusion:**

MCP-1 and IL-8 are independent prognostic biomarkers in AIS patients treated with intravenous thrombolysis. Their sustained elevation and combined use in a clinical prediction nomogram significantly enhance early risk stratification, offering a potential tool for personalized management.

## Introduction

Acute ischemic stroke (AIS) accounts for approximately 85% of all strokes worldwide and remains a leading cause of long-term disability and mortality despite advances in reperfusion therapies (1). Beyond the initial ischemic insult, neuroinflammation plays a central role in secondary brain injury and functional recovery (2). Following cerebral ischemia, damaged neurons and glial cells release damage-associated molecular patterns (DAMPs), triggering activation of resident microglia and recruitment of peripheral immune cells across the compromised blood–brain barrier (3). This inflammatory response, while initially protective, can become maladaptive and exacerbate tissue damage.

Among the myriad inflammatory mediators, monocyte chemoattractant protein-1 (MCP-1/CCL2) and interleukin-8 (IL-8/CXCL8) have emerged as key players. MCP-1 recruits monocytes/macrophages to ischemic lesions (4), and elevated levels have been associated with larger infarct volumes and worse neurological outcomes (5). IL-8 serves as a primary chemoattractant for neutrophils (6), and its elevation in the acute phase of stroke has been independently linked to poor functional outcomes and increased mortality (7).

Despite these established associations, several gaps remain. First, most studies rely on single-point measurements at admission, failing to capture the dynamic evolution of these chemokines during the critical first 72 h. Second, whether specific stroke etiologies (TOAST classification) or infarct locations differentially activate these pathways remains unclear. Third, there is a lack of practical prognostic tools that integrate these biomarkers with established clinical predictors for early risk stratification.

The primary aim of this study was to develop and temporally validate an early prediction model for 90-day poor outcome in AIS patients who received intravenous thrombolysis using admission-available clinical variables and the inflammatory biomarkers MCP-1 and IL-8. As secondary analyses, we further explored the early temporal trajectories of these biomarkers and their associations with stroke subtype and infarct location to provide biological context for the prognostic model.

## Materials and methods

### Study design and participants

This retrospective observational study was conducted at Liaocheng People’s Hospital. We systematically screened the electronic medical records of all patients diagnosed with AIS who were admitted within 4.5 h of symptom onset and received intravenous thrombolysis between September 2023 and October 2024. This study was therefore specifically conducted in the context of intravenous thrombolysis, and the developed prognostic model is intended for early risk stratification in this treatment-defined population, not for all AIS patients without thrombolysis.

This study was conducted in accordance with the principles of the Declaration of Helsinki. The research protocol was reviewed and approved by the Ethics Committee of Liaocheng People’s Hospital (approval no. 2024037). Due to the retrospective nature of the study and the use of anonymized clinical data and archived samples that were collected during routine clinical care, the requirement for individual informed consent was waived by the ethics committee.

### AIS cohort

This group consisted of 300 patients diagnosed with AIS who received intravenous thrombolysis and were admitted to Liaocheng People’s Hospital between September 2023 and October 2024. Eligible patients met the following criteria: (i) age ≥18 years; (ii) clinical diagnosis of AIS confirmed by diffusion-weighted magnetic resonance imaging (DWI-MRI) or computed tomography (CT) upon admission; (iii) presentation within 4.5 h of symptom onset and receipt of intravenous alteplase; (iv) availability of a sample collected at admission (prior to thrombolysis); and (v) complete 90-day follow-up data with a documented modified Rankin Scale (mRS) score. Patients with hemorrhagic stroke, subarachnoid hemorrhage, stroke mimics, concurrent severe systemic infection, active malignancy, autoimmune/inflammatory disorders, use of immunosuppressants or long-term corticosteroids, pregnancy, lactation, or missing essential data were excluded.

A separate healthy control group (*n* = 100) was included for descriptive reference only; these individuals were free of stroke and major chronic diseases. As the prognostic model was developed exclusively within the AIS cohort, the healthy control comparison was not used for model building or validation.

To perform a temporal validation of the prognostic model, the 300 AIS patients were sorted by admission date (from earliest to latest). The first 200 patients were assigned to the development cohort, and the subsequent 100 patients to the temporal validation cohort. Baseline characteristics were compared between the two cohorts to ensure no significant differences.

To ensure the final prognostic model is suitable for early risk stratification (i.e., at the time of admission or prior to thrombolysis), we restricted candidate predictors to those routinely available within the first hours of presentation. These included: demographics (age, sex), admission NIHSS score, infarct volume measured on admission DWI-MRI, onset-to-treatment time (OTT), standard laboratory parameters (FPG, WBC, PLT, etc.), and MCP-1 and IL-8 levels. Post-admission events such as cerebral edema and symptomatic hemorrhage, while prognostically important, were excluded from the final model because they cannot be known at the initial decision point. These variables were only included in a separate exploratory analysis to describe their association with outcome.

**Figure 1:**
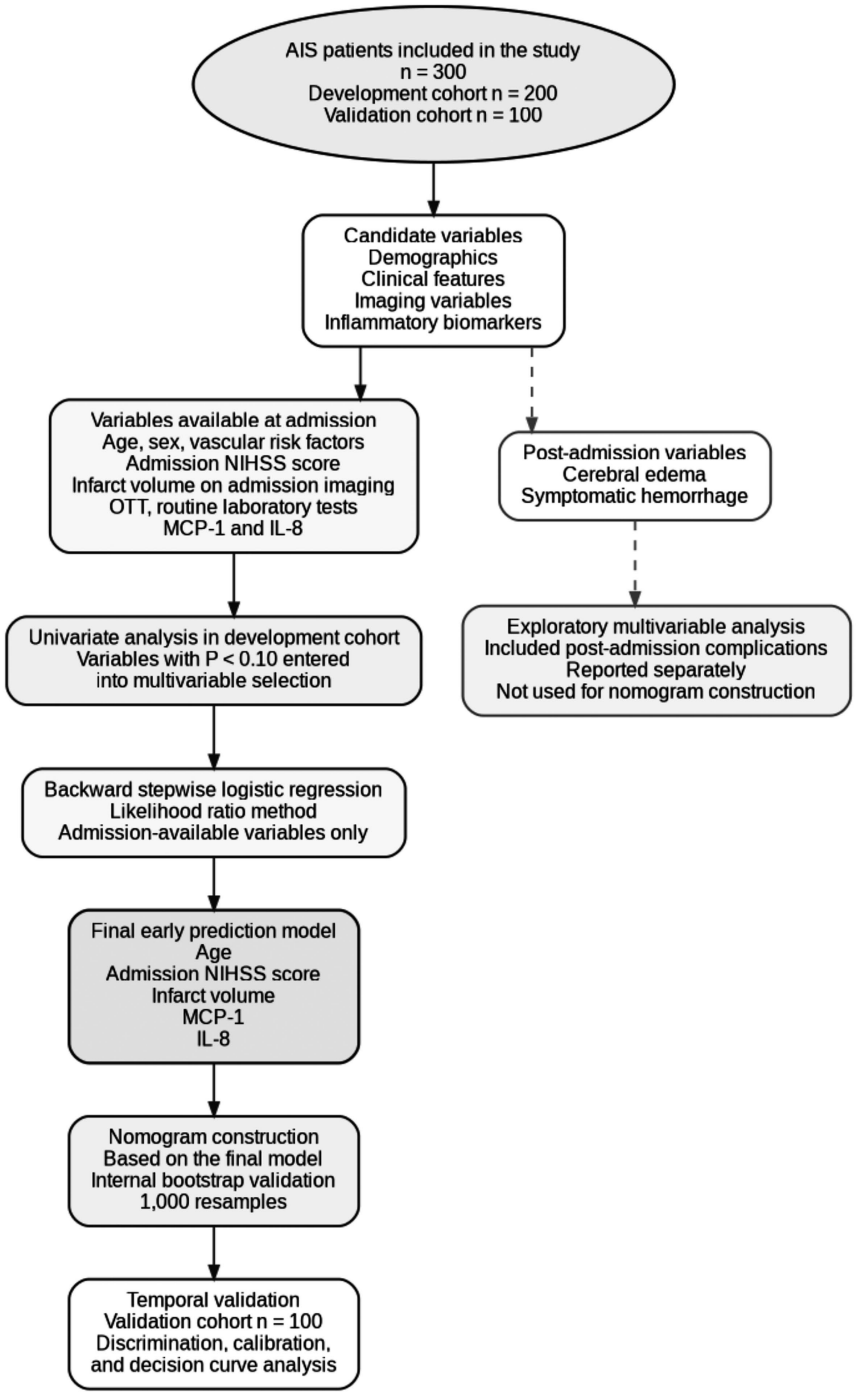
Variable selection and model development flowchart.

The stepwise selection process and the distinction between admission-available variables and post-admission complications are summarized in Figure 1.

It is important to emphasize that all patients in the AIS cohort met the eligibility criteria of receiving intravenous alteplase within 4.5 h of symptom onset. Consequently, the prognostic model developed in this study is strictly applicable only to AIS patients undergoing intravenous thrombolysis. The model is not intended for, and has not been validated in, AIS patients treated with mechanical thrombectomy alone, those receiving no reperfusion therapy, or those with onset-to-treatment time beyond 4.5 h. This treatment-defined scope should be explicitly recognized when interpreting or applying the model.

### Sample size determination

A total of 322 consecutive patients were initially identified. After applying strict eligibility criteria, 300 patients with complete datasets were included in the final analysis. This sample size was determined based on the availability of eligible cases with all required data points during the study period. No formal *a priori* sample size calculation was performed because this was a retrospective study using all eligible consecutive cases within the predefined time window. To assess the precision of the estimated model parameters, we calculated the 95% confidence intervals for the odds ratios and the C-index. The development cohort contained 73 poor-outcome events, which, according to the rule of thumb of 10–15 events per candidate predictor, would support the inclusion of approximately 5–7 predictors in the final model. Our final model included 5 predictors, which is within this range.

### Clinical data and sample retrieval

All clinical data, including demographics, medical history, laboratory results, imaging reports, and 90-day mRS scores, were retrospectively extracted from the hospital’s clinical databases. Admission samples, which had been routinely collected, processed, and archived as part of standard clinical care, were retrieved from the hospital’s biobank for biomarker analysis.

### Prognostic grouping

Patients were stratified into two groups based solely on their 90-day functional outcome, as documented in the medical records:

- Favorable outcome group (Group A, *n* = 192): mRS score ≤2.
- Poor outcome group (Group B, *n* = 108): mRS score >2.

### Detection of MCP-1 and IL-8

(1) Sample collection and processing: At hospital admission, 2 mL of venous EDTA-anticoagulated blood and 3 mL of coagulated blood were collected from each patient for routine clinical laboratory tests, without causing any additional trauma to the subjects. After clinical testing, the remaining blood samples were anonymized and processed for research purposes. The use of these residual samples for biomarker analysis was covered by the ethics approval with waiver of individual informed consent, as the samples were de-identified prior to analysis. Anticoagulated blood samples were centrifuged at 3500 r/min for 10 minutes (radius = 8 cm). The supernatant plasma was carefully collected, aliquoted, and stored at -80°C until MCP-1 and IL-8 levels were measured. (2) Detection of MCP-1 and IL-8: MCP-1 and IL-8 levels were measured using the Luminex multiplex fluorescence assay kit (R&D Systems, USA) according to the manufacturer’s instructions, and the differences between the observation and control groups were compared. (3) Standardization and quality control: During the collection and testing of laboratory indicators, we followed standardized sampling and handling procedures to ensure data consistency and comparability. All samples were analyzed using identical instruments and methods to minimize experimental error. In addition, all laboratory data underwent strict quality control (QC) and quality assurance (QA) procedures to guarantee the accuracy and reliability of the results. (4) Dynamic Monitoring and Ratio Calculation: For the 150-patient substudy cohort, the separation and Luminex assay procedures were identical for all three time points to ensure comparability. The delta values (*Δ*) for each marker were calculated as the difference between T3 and T1 levels (Δ = T3–T1).

All samples from AIS patients (including the development cohort, temporal validation cohort, and dynamic substudy) and healthy controls were analyzed in a single batch using the same Luminex assay kit lot (R&D Systems, USA) to eliminate inter-batch variability. The assays were performed consecutively over a period of 5 working days. Samples from AIS patients and healthy controls were interleaved across the assay plates to avoid systematic bias. The laboratory technician was blinded to the outcome status (mRS) and to the group assignment (AIS vs. control) during the entire testing process. The archived samples had been stored at −80 °C for a maximum of 14 months prior to analysis, and all samples underwent the same number of freeze–thaw cycles (one cycle only). No sample showed visible hemolysis or turbidity.

### Clinical data collection and outcome definition

Collected variables included: Demographics: age, sex; Lifestyle factors: smoking, alcohol consumption; Medical history: hypertension, diabetes, atrial fibrillation, prior stroke; Complications: intracranial edema, symptomatic hemorrhage; Radiological data: infarct volume, Trial of Org 10,172 in Acute Stroke Treatment (TOAST) classification (8), responsible vessel; Neurological assessment: National Institutes of Health Stroke Scale (NIHSS) score at admission (9).

#### Treatment timing: onset-to-treatment time (OTT)

##### Laboratory parameters

Fasting plasma glucose (FPG), creatinine (Scr), hemoglobin (Hb), white blood cell count (WBC), platelet count (PLT), prothrombin time (PT), activated partial thromboplastin time (APTT), fibrinogen (FIB), total cholesterol (TC), triglyceride (TG), low-density lipoprotein cholesterol (LDL-C), and high-density lipoprotein cholesterol (HDL-C).

##### Functional outcome

Assessed using the modified Rankin Scale (mRS) (10) at 90 days after onset. Follow-up was conducted via outpatient visits or telephone interviews using standardized questionnaires by trained assessors to ensure consistency. Patients with mRS ≤ 2 were defined as the good outcome group, and those with mRS > 2 as the poor outcome group.

##### Radiological assessment

Measurements were performed using UIH intelligent analysis software, with manual correction by two blinded neuroradiologists. The TOAST classification and infarct location (anterior circulation territories including ICA, MCA, ACA; posterior circulation territories including VA, BA) were adjudicated by a stroke neurologist and a neuroradiologist based on all available clinical and imaging data.

### Experimental quality control and reproducibility assurance

1. All laboratory procedures were performed by the same trained technician to ensure procedural consistency.
2. All instruments were calibrated and performance-verified before analysis.
3. sample collection, storage, and detection followed a standardized operating procedure (SOP) with detailed process records.
4. All raw fluorescence intensity values and calculated concentrations were cross-checked by two investigators and independently verified by a third-party statistician.
5. To ensure objectivity, all data were anonymized before analysis, and analysts were blinded to group allocation.
6. Experimental and clinical data were entered into a secure, encrypted database with tiered access permissions to ensure data integrity and confidentiality.

### Statistical analysis

Statistical analysis was performed using SPSS 26.0 (IBM, USA), GraphPad Prism 9.0 (GraphPad Software, USA), and R software (version 4.3.1, R Foundation for Statistical Computing). Continuous variables are presented as mean ± standard deviation (SD) for normally distributed data or median (interquartile range) for skewed data, and categorical variables as frequencies (percentages). Normality was assessed using the Shapiro–Wilk test.

#### Group comparisons

Intergroup differences were analyzed using independent-sample *t*-tests, Mann–Whitney U tests, one-way ANOVA (with Tukey’s *post-hoc* test), or χ^2^ tests, as appropriate.

#### Temporal dynamics analysis

For the sub-study cohort, repeated measures of MCP-1 and IL-8 were analyzed using linear mixed-effects models (LMM) with *group* (good vs. poor prognosis), *time*, and their *interaction* as fixed effects, and subject ID as a random effect. This accounts for within-subject correlation.

To construct a model suitable for early risk stratification, we performed multivariate logistic regression using only variables available at admission. All candidate predictors (age, sex, smoking, drinking, hypertension, diabetes, atrial fibrillation, prior stroke, admission NIHSS score, infarct volume, OTT, FPG, Scr, Hb, WBC, PLT, PT, APTT, FIB, TC, TG, LDL-C, HDL-C, MCP-1, IL-8) were first assessed in univariate analysis. Candidate predictors were prespecified based on clinical relevance, prior literature, and availability at the time of admission. Given the development cohort contained only 73 poor-outcome events, we limited the number of predictors in the final multivariate model by applying a conservative variable selection strategy: only variables with a univariate *p*-value <0.10 were considered for entry, and backward stepwise selection (with AIC criterion) was used to obtain a parsimonious model. The final model was intentionally restricted to 5 predictors (age, NIHSS, infarct volume, MCP-1, IL-8) to maintain an events-per-variable ratio of approximately 14.6:1, which is above the commonly recommended minimum of 10:1 for reliable logistic regression. The final model was intentionally restricted to a parsimonious set of admission-available predictors for nomogram construction rather than to maximize statistical fit. To describe the prognostic impact of post-admission events, we performed a separate multivariate analysis that additionally included cerebral edema and symptomatic hemorrhage. These results are presented for completeness but do not inform the early prediction model.

A nomogram was constructed based on the early-risk prediction model (age, NIHSS score, infarct volume, MCP-1, IL-8) using the rms package in R. Post-admission complications were excluded from the nomogram because they are not available at the time of initial risk stratification. The model’s discriminative ability was quantified by the concordance index (C-index), equivalent to the area under the ROC curve (AUC). Internal validation was performed using 1,000 bootstrap resamples within the development cohort to correct for optimism and obtain a bias-corrected C-index. Calibration (agreement between predicted and observed probabilities) was assessed using the Hosmer-Lemeshow goodness-of-fit test and calibration plots.

For temporal validation, the nomogram was applied to the validation cohort without refitting. Discrimination was assessed by calculating the C-index and its 95% confidence interval. Calibration was evaluated by plotting predicted probabilities against observed outcomes and performing the Hosmer-Lemeshow test. Decision curve analysis was repeated in the validation cohort to assess net clinical benefit.

Decision curve analysis (DCA) was performed using the DCA package in R to evaluate the net clinical benefit of the prediction models across a range of threshold probabilities (10–70%). Net benefit was calculated to compare four strategies: (1) treat all patients as high-risk, (2) treat none, (3) use a clinical-only model incorporating the three core clinical predictors available at admission (age, NIHSS score, infarct volume), and (4) use the combined model (clinical predictors + MCP-1 + IL-8). This comparison directly quantifies the incremental value of adding inflammatory biomarkers to the baseline clinical assessment that is available at the time of early risk stratification.

#### ROC curve comparisons

The predictive performances of MCP-1, IL-8, their combination were evaluated using ROC curves. The “combination” was derived from the predicted probabilities of a logistic regression model containing both MCP-1 and IL-8. Differences between AUCs were compared using the DeLong test. The optimal cut-off value for each biomarker was determined by maximizing the Youden’s index (J = sensitivity + specificity − 1).

A two-sided *p* value < 0.05 was considered statistically significant.

## Results

### Comparison of MCP-1 and IL-8 levels

MCP-1 and IL-8 levels were significantly higher in the AIS cohort than in healthy controls (both *p* < 0.05). MCP-1 and IL-8 levels in the good outcome group were 196.84 ± 49.21 pg./mL and 72.36 ± 15.42 pg./mL, respectively, while those in the poor outcome group were 246.79 ± 57.38 pg./mL and 88.54 ± 17.73 pg./mL, respectively. Both markers were significantly lower in the good outcome group than in the poor outcome group (*p* < 0.05).

### Comparison of clinical data between good and poor outcome groups

As shown in Table 1, there were significant differences between the good outcome group and poor outcome group in age, hypertension, atrial fibrillation, cerebral edema, symptomatic hemorrhage, infarct volume, NIHSS score, OTT, FPG, WBC, and PLT levels (all *p* < 0.05). There were no statistically significant differences between groups in the remaining indicators (gender, smoking history, drinking history, diabetes, previous stroke history, TOAST classification, responsible vessel, Scr, Hb, PT, APTT, FIB, TC, TG, LDL-C, HDL-C) (all *p* > 0.05).

**Table 1:** Comparison of clinical data between good outcome group and poor outcome group (*x̅* ± s, *n* [%]).

Clinical data	Good outcome group (*n* = 192)	Poor outcome group (*n* = 108)	*t/x^2^*	*p*
Age (years)	61.42 ± 10.53	70.57 ± 11.29	7.037	<0.001
Gender	–	–	0.132	0.715
Male	115 (59.90%)	67 (62.04%)	–	–
Female	77 (40.10%)	41 (37.96%)	–	–
Smoking history	79 (41.15%)	51 (47.22%)	1.039	0.308
Drinking history	73 (38.02%)	46 (42.59%)	0.603	0.437
Hypertension	95 (49.48%)	75 (69.44%)	11.220	<0.001
Diabetes	48 (25.00%)	37 (34.26%)	2.918	0.087
Atrial fibrillation	28 (14.58%)	30 (27.78%)	7.715	0.005
Previous stroke	24 (12.50%)	19 (17.59%)	1.459	0.226
Cerebral edema	17 (8.85%)	24 (22.22%)	10.468	0.001
Symptomatic hemorrhage	9 (4.69%)	17 (15.74%)	10.668	0.001
Infarct volume (mL)	21.52 ± 6.46	30.87 ± 8.53	10.690	<0.001
TOAST	–	–	2.375	0.123
Atherosclerosis	124 (64.58%)	60 (55.56%)	–	–
Cardioembolic	46 (23.96%)	37 (34.26%)	–	–
Others	22 (11.46%)	11 (10.19%)	–	–
Responsible vessel	–	–	0.042	0.836
ICA/MCA/ACA	132 (68.75%)	73 (67.59%)	–	–
VA/BA	38 (19.79%)	25 (23.15%)	–	–
Tandem lesion	22 (11.46%)	10 (9.26%)	–	–
Admission NIHSS score (points)	7.82 ± 3.41	13.65 ± 4.72	12.330	<0.001
OTT (h)	2.38 ± 0.89	3.25 ± 1.12	7.389	<0.001
FPG (mmol/L)	5.89 ± 1.14	6.62 ± 1.36	4.960	<0.001
Scr (μmol/L)	73.26 ± 14.58	75.84 ± 15.41	1.441	0.150
Hb (g/L)	132.41 ± 15.83	130.27 ± 16.54	1.105	0.269
WBC (×10^9^/L)	6.72 ± 1.81	8.37 ± 2.05	7.221	<0.001
PLT (×10^9^/L)	204.53 ± 47.25	181.72 ± 51.38	3.888	<0.001
PT (s)	12.31 ± 0.93	12.46 ± 0.97	1.320	0.187
APTT (s)	31.42 ± 3.71	32.06 ± 4.23	1.362	0.174
FIB (g/L)	3.47 ± 0.86	3.59 ± 0.92	1.131	0.258
TC (mmol/L)	4.63 ± 0.72	4.58 ± 0.81	0.551	0.581
TG (mmol/L)	1.43 ± 0.51	1.47 ± 0.56	0.629	0.529
LDL-C (mmol/L)	2.84 ± 0.69	2.91 ± 0.75	0.817	0.414
HDL-C (mmol/L)	1.12 ± 0.31	1.08 ± 0.29	1.097	0.273

### Temporal dynamics of inflammatory markers

**Figure 2:**
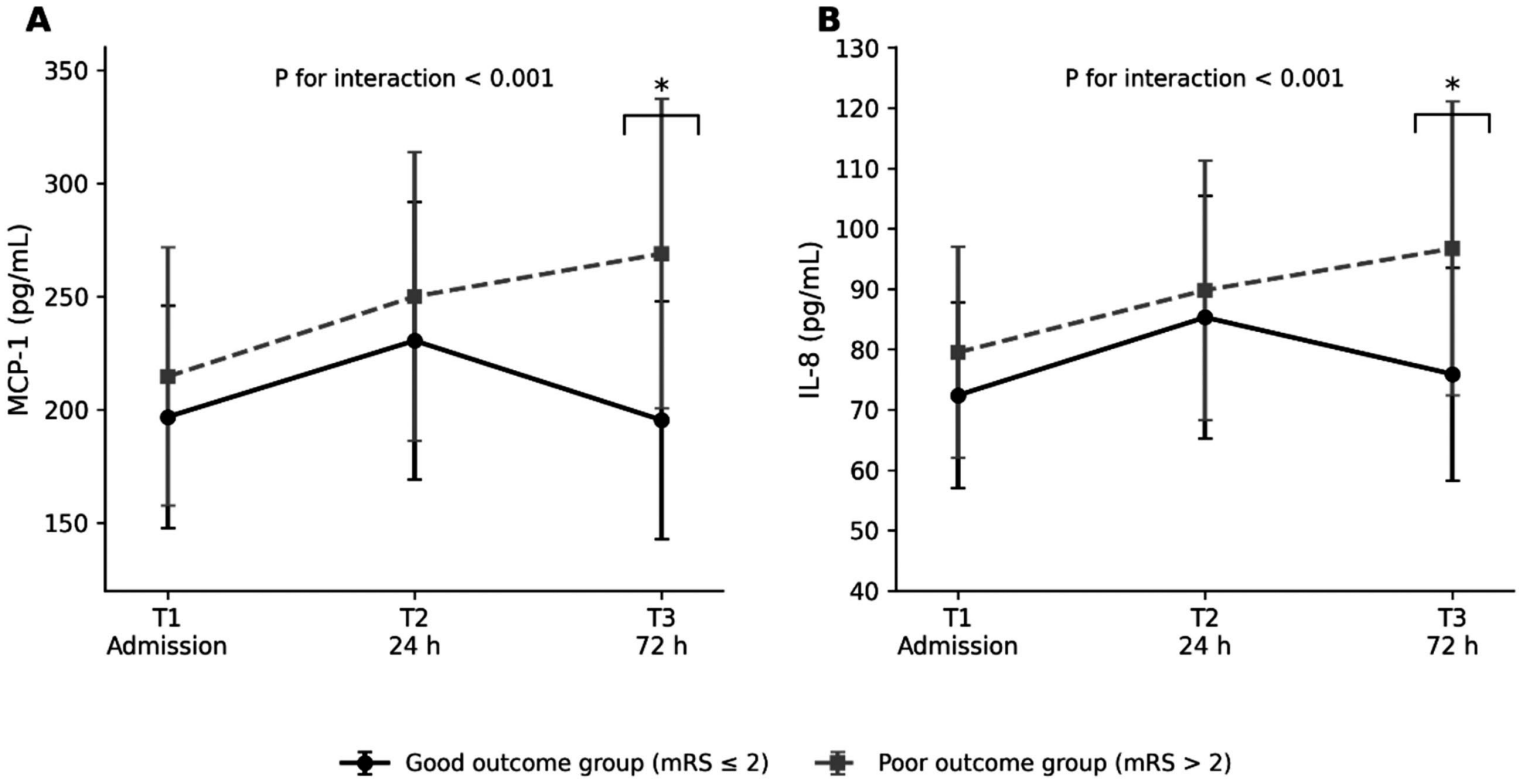
Temporal profiles of MCP-1 **(A)** and IL-8 **(B)** levels in AIS patients with good and poor outcomes. Serial measurements were taken at admission (T1), 24 h (T2), and 72 h (T3) post-thrombolysis in a sub-study cohort (*n* = 150). Patients were stratified by 90-day mRS scores into good outcome (mRS ≤ 2) and poor outcome (mRS > 2) groups. Data are presented as mean ± SD. Group × time interactions were assessed using linear mixed-effects models.

To capture the evolution of inflammatory responses, we performed serial measurements in a subset of 150 patients at three time points: admission (T1), 24 h post-thrombolysis (T2), and 72 h (T3). As illustrated in Figure 2, both MCP-1 and IL-8 displayed distinct temporal patterns that differed significantly between patients with good (mRS ≤ 2) and poor (mRS > 2) outcomes (*p* for interaction <0.001).

In the good prognosis group, MCP-1 levels peaked at T2 (230.5 ± 61.2 pg./mL) and subsequently declined by T3 (195.4 ± 52.7 pg./mL), approaching baseline. In contrast, the poor prognosis group exhibited a sustained elevation, with MCP-1 levels continuing to rise at T3 (268.9 ± 68.5 pg./mL). Similarly, IL-8 levels in the good prognosis group showed a transient increase at T2 (85.3 ± 20.1 pg./mL) followed by resolution, while the poor prognosis group demonstrated progressive elevation throughout the observation period (T3: 96.7 ± 24.3 pg./mL).

Notably, the ΔMCP-1 (T3-T1) and ΔIL-8 (T3-T1)—representing the change from baseline to 72 h—were significantly greater in the poor prognosis group (54.3 ± 22.7 pg./mL vs. −12.8 ± 18.4 pg./mL for MCP-1, 17.2 ± 9.8 pg./mL vs. 3.5 ± 7.2 pg./mL for IL-8, both *p* < 0.001).

### Exploratory analysis

The TOAST classification includes five etiological categories. For this exploratory subgroup analysis, we included only large-artery atherosclerosis, cardioembolism, and other determined etiology. The small vessel occlusion subtype was excluded because no patient in our thrombolyzed AIS cohort met the criteria for this subtype (all patients had moderate-to-severe neurological deficits requiring reperfusion therapy, and small vessel strokes were typically managed without thrombolysis in our center). The undetermined etiology subtype was excluded due to its heterogeneous nature, incomplete diagnostic workup in many cases, and the resulting difficulty in attributing chemokine levels to a specific pathophysiological mechanism. Thus, the analysis was restricted to the three subgroups with clear etiological definitions and sufficient sample size in our cohort. As shown in Table 2, MCP-1 and IL-8 levels differed significantly among these three subtypes (*p* < 0.05). Patients with cardioembolic stroke had numerically higher levels of MCP-1 (245.3 ± 62.1 pg./mL) and IL-8 (85.7 ± 20.3 pg./mL) compared to those with large-artery atherosclerosis (220.8 ± 55.4 pg./mL and 80.2 ± 18.9 pg./mL, respectively) and other determined etiology (198.6 ± 48.7 pg./mL and 72.5 ± 16.8 pg./mL). Post-hoc comparisons showed that the cardioembolic group had higher MCP-1 and IL-8 levels than both the atherosclerotic group (*p* = 0.003 for MCP-1; *p* = 0.012 for IL-8) and the other etiology group (*p* < 0.001 for both). However, these differences may be confounded by stroke severity (e.g., NIHSS score, infarct volume), which also varied across etiological subtypes. Therefore, these findings should be considered exploratory and hypothesis-generating. Supplementary Table S1 provides adjusted analyses accounting for age, NIHSS score, infarct volume, and vascular risk factors; After adjustment, the association between cardioembolic stroke and MCP-1/IL-8 levels was no longer statistically significant, suggesting confounding by stroke severity. Anterior circulation remained associated with higher IL-8 levels, though the effect size was attenuated.

**Table 2:** Exploratory analysis: MCP-1 and IL-8 levels according to TOAST classification (unadjusted).

TOAST subtype	*n* (%)	MCP-1 (pg/mL)	IL-8 (pg/mL)	*p* value
Large-artery atherosclerosis	184 (61.3%)	220.8 ± 55.4	80.2 ± 18.9	Reference
Cardioembolism	83 (27.7%)	245.3 ± 62.1^*†^	85.7 ± 20.3^*†^	<0.001
Other determined etiology	33 (11.0%)	198.6 ± 48.7	72.5 ± 16.8	0.024
Overall *p* value		<0.001	0.001	

*p* < 0.05 vs. large-artery atherosclerosis group; ^†*^p < 0.05 vs. other etiology group. The analysis included only three of the five TOAST categories. Small vessel occlusion and undetermined etiology were excluded for reasons stated in the methods (exploratory analysis).

We also performed an exploratory analysis to assess whether infarct location was associated with inflammatory biomarker levels. As presented in Table 3, patients with anterior circulation strokes (involving ICA, MCA, or ACA territories) had numerically higher IL-8 levels compared to those with posterior circulation strokes (VA/BA territories) (81.5 ± 19.2 pg./mL vs. 73.8 ± 16.5 pg./mL, *p* = 0.008). MCP-1 levels did not differ significantly between these groups (219.4 ± 57.6 pg./mL vs. 213.2 ± 54.3 pg./mL, *p* = 0.412). Among anterior circulation strokes, MCA territory infarcts showed the highest IL-8 levels (83.7 ± 20.1 pg./mL). These observations are descriptive and unadjusted for potential confounders such as infarct volume, which is often larger in anterior circulation strokes. Accordingly, they should be interpreted as preliminary and warrant further investigation.

**Table 3:** Exploratory analysis: inflammatory marker levels by infarct location (unadjusted).

Infarct location	*n*	MCP-1 (pg/mL)	*p* value	IL-8 (pg/mL)	*p* value
Anterior circulation	205	219.4 ± 57.6	0.412	81.5 ± 19.2	0.008
ICA territory	42	215.8 ± 56.2		79.8 ± 18.4	
MCA territory	136	221.3 ± 58.7		83.7 ± 20.1	
ACA territory	27	214.6 ± 52.9		76.3 ± 17.8	
Posterior circulation	63	213.2 ± 54.3	Reference	73.8 ± 16.5	Reference
VA territory	38	210.5 ± 53.1		74.2 ± 17.1	
BA territory	25	217.1 ± 56.4		73.2 ± 15.8	
Tandem/Other	32	208.9 ± 52.7	0.689	75.6 ± 17.3	0.103

### Baseline characteristics of development and validation cohorts

As shown in Supplementary Table S2, there were no significant differences between the development cohort (*n* = 200) and temporal validation cohort (*n* = 100) in age, sex, vascular risk factors, NIHSS score, infarct volume, or MCP-1 and IL-8 levels (all *p* > 0.05). The proportion of patients with poor outcome (mRS > 2) was 36.5% in the development cohort and 35.0% in the validation cohort (*p* = 0.793).

### Multivariate analysis for early risk stratification

Using variables available at admission, multivariate logistic regression identified age, NIHSS score, infarct volume, MCP-1, and IL-8 as independent predictors of poor 90-day outcome (all *p* < 0.05, Table 4). These five variables were selected for nomogram construction.

**Table 4:** Multivariate logistic regression analysis of variables available at admission for predicting poor 90-day outcome (development cohort, *n* = 200).

Variable	β	SE	Wald *χ*^2^	*p*	OR	95% CI
Age	0.047	0.012	15.3	<0.001	1.048	1.024–1.073
Admission NIHSS score	0.219	0.048	20.83	<0.001	1.245	1.133–1.368
Infarct volume	0.081	0.021	14.96	<0.001	1.084	1.041–1.129
MCP-1	0.012	0.004	9.22	0.002	1.012	1.004–1.021
IL-8	0.025	0.008	9.77	0.002	1.025	1.009–1.041

Variables included in the multivariate model: age, sex, hypertension, diabetes, atrial fibrillation, prior stroke, admission NIHSS score, infarct volume, OTT, FPG, WBC, PLT, MCP-1, IL-8.

### Exploratory analysis of post-admission complications

When post-admission complications (cerebral edema, symptomatic hemorrhage) were added to the model in an exploratory analysis, they also showed strong independent associations with poor outcome (cerebral edema: OR 2.52, 95% CI 1.32–4.79, *p* = 0.005; symptomatic hemorrhage: OR 3.12, 95% CI 1.32–7.38, *p* = 0.010). However, these variables are not available at admission and therefore were not included in the final early prediction model (Supplementary Table S3).

### Predictive value of MCP-1, IL-8, and their combination for short-term poor prognosis in AIS patients

**Figure 3:**
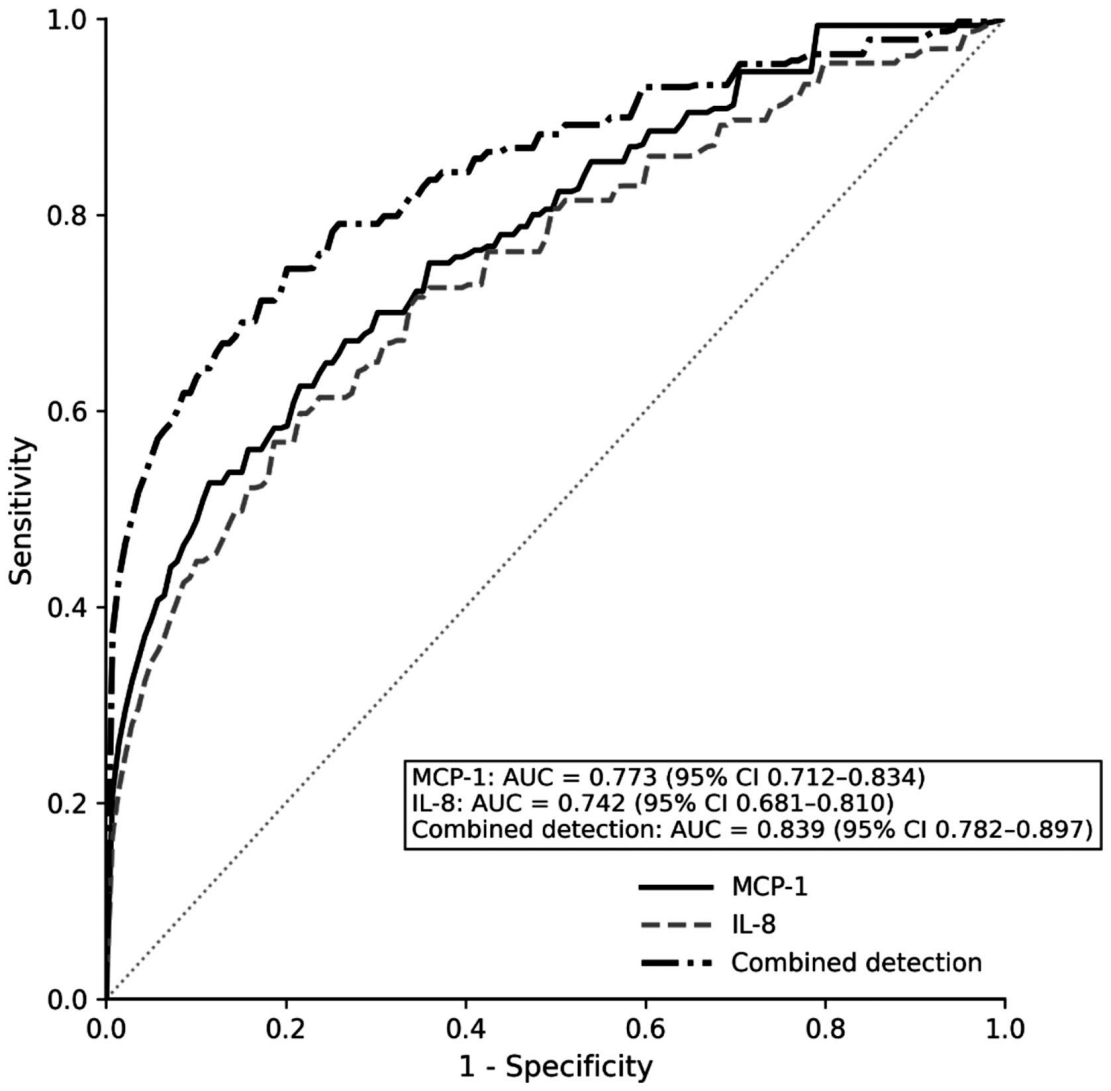
Receiver operating characteristic (ROC) curves of MCP-1, IL-8, and their combination for predicting poor outcome in AIS patients. ROC, receiver operating characteristic; AUC, area under the curve; CI, confidence interval; MCP-1, monocyte chemoattractant protein-1; IL-8, interleukin-8; AIS, acute ischemic stroke; mRS, modified Rankin Scale. ROC analysis was performed in the development cohort (*n* = 200). The combined model was derived from logistic regression including both biomarkers. The diagonal dashed line represents the reference line (AUC = 0.5).

ROC analysis was performed in the development cohort (*n* = 200). The poor outcome group was defined as mRS > 2 at 90 days (*n* = 73), and the good outcome group as mRS ≤ 2 (*n* = 127). The combined detection model was derived from logistic regression including both MCP-1 and IL-8 as continuous variables. The AUC with 95% confidence intervals was: MCP-1: 0.773 (0.712–0.834); IL-8: 0.742 (0.681–0.810); combined detection: 0.839 (0.782–0.897). Differences between AUCs were compared using the DeLong test. The combined model showed significantly better predictive performance than either single marker (*p* < 0.05 for both comparisons). The diagonal dashed line represents the reference line (AUC = 0.5). The combined diagnostic performance was significantly superior to either single indicator (Zcombined–MCP-1 = 6.836, Zcombined–IL-8 = 7.224, *p* < 0.05) (Table 5; Figure 3).

**Table 5:** Comparison of the predictive efficiency of MCP-1, IL-8, and combined detection for short-term poor prognosis in AIS patients.

Indicator	Cut-off value	AUC	95% CI	Sensitivity (%)	Specificity (%)	Youden index
MCP-1	225.60 pg./mL	0.773	0.712–0.834	74.1	72.3	0.464
IL-8	80.50 pg./mL	0.742	0.681–0.810	70.4	70.1	0.405
Combined detection	–	0.839	0.782–0.897	81.5	78.3	0.597

To assess the added prognostic value of MCP-1 and IL-8 beyond readily available clinical predictors, we constructed a clinical-only model using age, admission NIHSS score, and infarct volume—the three clinical variables that were significant in multivariate analysis and are commonly available at admission. In the development cohort, the clinical-only model yielded an AUC of 0.801 (95% CI: 0.741–0.861). Adding MCP-1 and IL-8 to this clinical baseline increased the AUC to 0.839 (95% CI: 0.782–0.897), representing an absolute improvement of 0.038. The Delong test comparing the two models showed statistical significance (*p* = 0.027). In the temporal validation cohort, the clinical-only model achieved an AUC of 0.798 (95% CI: 0.726–0.870), while the combined model (clinical + biomarkers) achieved an AUC of 0.849 (95% CI: 0.766–0.916), again showing an incremental gain (ΔAUC = 0.051). These results suggest that while the clinical variables alone provide reasonable discrimination, the addition of MCP-1 and IL-8 offers a modest but statistically significant improvement in prognostic accuracy.

### Development of a clinical prediction nomogram

**Figure 4:**
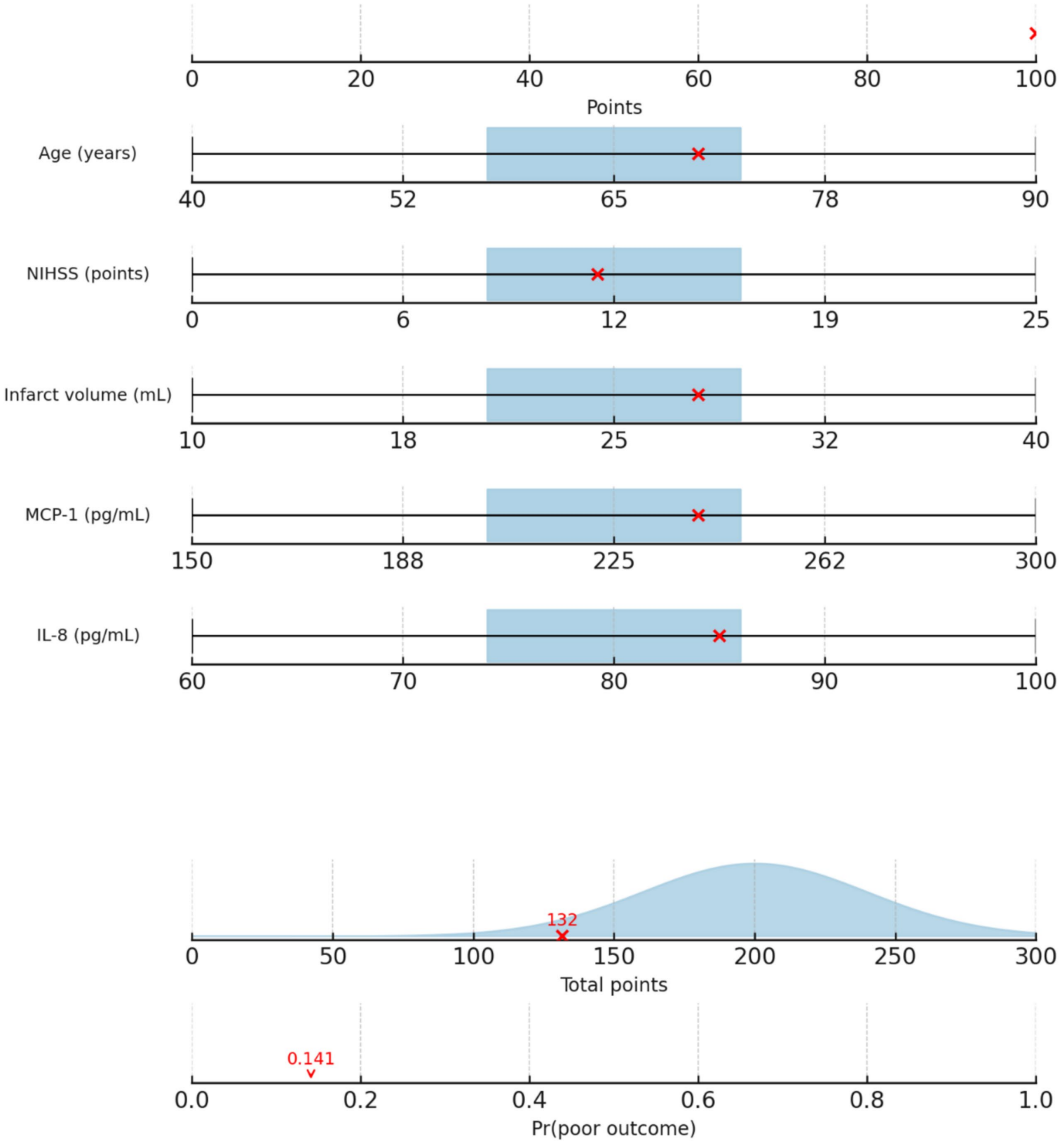
Nomogram for predicting 90-day poor outcome in AIS patients. The nomogram was developed using the development cohort (*n* = 200) and incorporates five independent predictors of poor outcome (mRS >2 at 90 days): age (years), admission NIHSS score (points), infarct volume (mL) MCP-1 (pg/mL), and IL-8 (pg/mL). Each variable is assigned a score on the Points scale (0–100).

To facilitate early clinical application, we constructed a nomogram (Figure 4) incorporating the five independent predictors identified in the multivariate analysis restricted to admission-available variables: age, NIHSS score, infarct volume, MCP-1, and IL-8. The nomogram demonstrated excellent discrimination, with a C-index of 0.872 (95% CI: 0.831–0.913) in the development cohort. Internal bootstrap validation (1,000 resamples) yielded a bias-corrected C-index of 0.851, indicating good internal consistency. Calibration plots showed good agreement between predicted and observed probabilities (Hosmer-Lemeshow test: *χ*^2^ = 7.24, *p* = 0.511).

### Temporal validation of the nomogram

**Figure 5:**
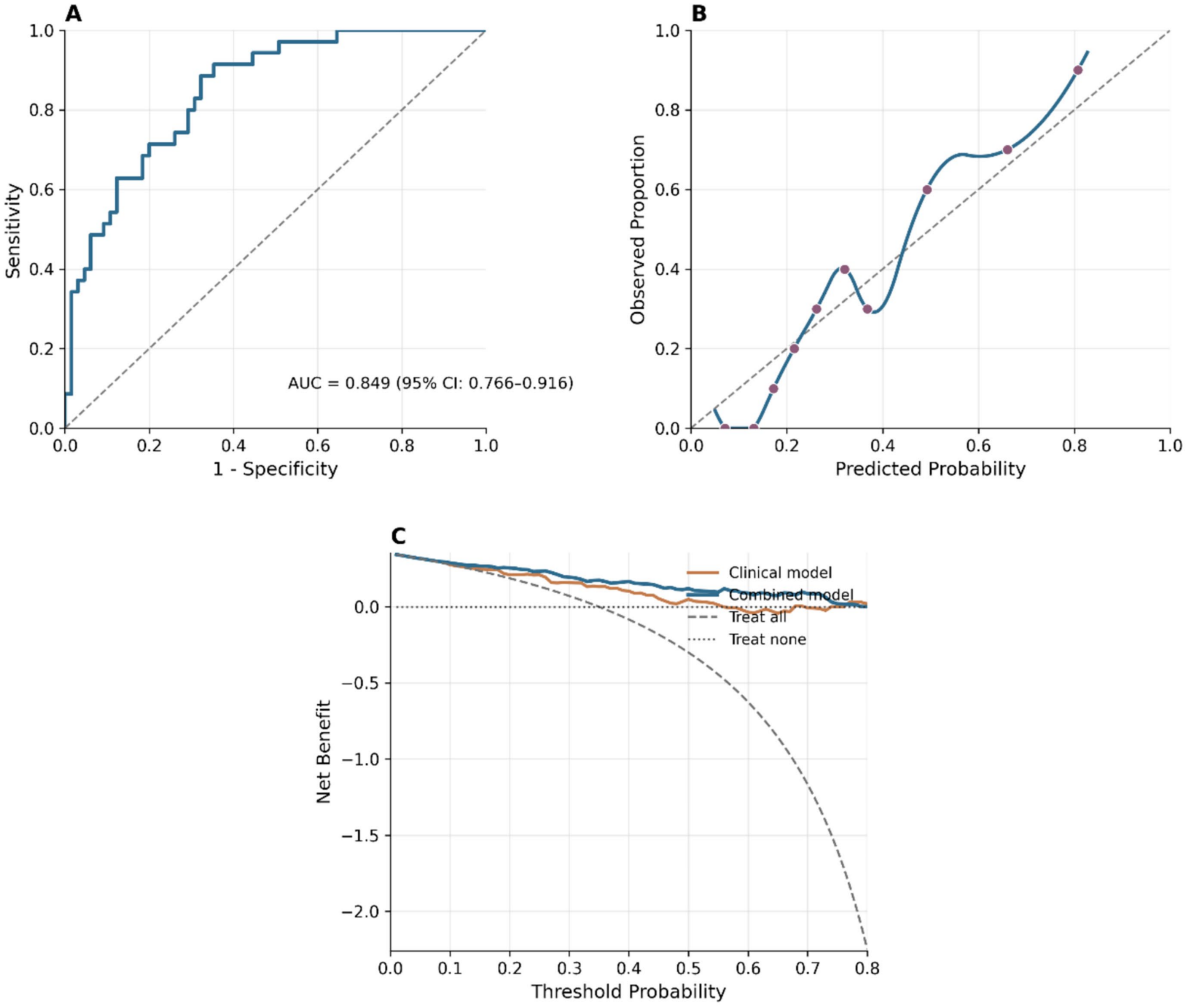
Temporal validation of the nomogram. **(A)** ROC curve in the temporal validation cohort (*n* = 100, subset of AIS patients) showing an AUC of 0.842. **(B)** Calibration plot showing agreement between predicted and observed probabilities in the validation cohort. **(C)** Decision curve analysis comparing the net benefit of the nomogram (combined model).

The nomogram was applied to the temporal validation cohort (*n* = 100) without modification. The C-index for predicting poor outcome was 0.849 (95% CI: 0.766–0.916), indicating good discriminative ability (Figure 5A). Calibration was satisfactory, with predicted probabilities closely matching observed outcomes (Hosmer-Lemeshow χ^2^ = 5.36, *p* = 0.718; Figure 5B). Decision curve analysis further supported the potential clinical utility of the nomogram in the validation cohort (Figure 5C). Overall, these findings support the temporal robustness of the model within the same institutional setting. The combined model shows the highest net benefit across threshold probabilities of 10–60%, indicating that incorporating MCP-1 and IL-8 improves clinical decision-making compared with using clinical factors alone.

### Clinical utility assessment by decision curve analysis

To further evaluate potential clinical usefulness, we performed decision curve analysis comparing four strategies in the validation cohort: treat all, treat none, a clinical-only model (age, admission NIHSS score, infarct volume), and the combined model incorporating MCP-1 and IL-8 in addition to clinical factors (Figure 5C). The combined model provided higher net benefit than the clinical-only model across clinically relevant threshold probabilities of approximately 10–60%. At a threshold probability of 30%, the net benefit of the combined model was 0.28, compared with 0.19 for the clinical-only model, corresponding to approximately 9 additional true-positive identifications per 100 patients without increasing false-positive classifications. These findings suggest that adding MCP-1 and IL-8 to conventional clinical predictors may improve decision support in early prognostic assessment, although the practical value of this improvement should be confirmed in external cohorts.

## Discussion

In this retrospective single-center study, we developed and temporally validated an admission-based prognostic model for 90-day functional outcome after acute ischemic stroke and found that MCP-1 and IL-8 added meaningful predictive information to conventional clinical variables. The resulting nomogram, which incorporated age, admission NIHSS score, infarct volume, MCP-1, and IL-8, demonstrated good discrimination and calibration, with supportive decision-curve findings in the temporal validation cohort. By design, the model was limited to variables available at the initial clinical evaluation, thereby prioritizing early applicability over maximal explanatory breadth. Importantly, all patients included in this study received intravenous thrombolysis, and therefore the model’s conclusions and performance should not be directly extrapolated to non-thrombolyzed AIS patients or those treated with mechanical thrombectomy alone. In parallel, the serial biomarker analysis showed that patients with poor outcome exhibited persistent chemokine elevation over the first 72 h, whereas those with good outcome showed a more transient inflammatory response. These dynamic findings do not constitute the primary predictive analysis, but they offer biological support for why admission MCP-1 and IL-8 may carry prognostic relevance in this setting.

Our findings are consistent with previous studies linking inflammatory chemokines to stroke outcomes. Prior work has shown that elevated MCP-1 levels correlate with larger infarct volumes, more severe neurological deficits, and increased risk of hemorrhagic transformation (5, 6), while elevated IL-8 levels have been associated with poor functional outcomes and increased mortality (7, 11). A recent nationwide registry study of 10,700 patients reported that low plasma MCP-1 levels were associated with reduced risks of all-cause mortality and poor functional outcome after ischemic stroke (12). Our study extends these observations by suggesting that the combination of both markers may improve predictive accuracy compared with either marker alone, possibly reflecting co-activation of both monocyte/macrophage and neutrophil axes (13, 14).

The improvement in AUC gained by adding MCP-1 and IL-8 to the clinical-only model (ΔAUC ≈ 0.04–0.05) is modest. Whether this magnitude of improvement translates into clinically meaningful reclassification at the individual patient level remains to be tested using net reclassification improvement (NRI) or integrated discrimination improvement (IDI) in future studies. Nevertheless, the decision curve analysis showed that the combined model provided higher net benefit than the clinical-only model across a range of threshold probabilities, supporting potential clinical utility despite the modest AUC increase.

A notable aspect of this study is the longitudinal observation that inflammation dynamics may carry prognostic information. Patients destined for good recovery exhibited a self-limiting inflammatory response, with chemokine levels peaking around 24 h and trending downward by 72 h. In contrast, patients with poor outcomes displayed sustained or escalating inflammatory states at the 72-h mark. This divergence raises the possibility that the capacity to resolve inflammation may influence recovery (15)—a hypothesis supported by recent work reporting evolving immune dysregulation in patients undergoing endovascular thrombectomy, where post-procedural elevations of IL-6 and adhesion molecules were associated with poorer outcomes (16).

The pathophysiological basis for sustained elevation may relate to thrombus composition and persistence. Experimental studies have shown that clots can induce monocyte gene expression, with IL-8 and MCP-1 among the upregulated transcripts; in such models, timely fibrinolysis attenuated this inflammatory response (17). This observation may help explain why patients with sustained inflammation at 72 h—potentially those with incomplete or delayed reperfusion—tended to have worse outcomes.

In exploratory analyses, we observed that patients with cardioembolic stroke had higher MCP-1 and IL-8 levels compared to those with large-artery atherosclerosis or other determined etiologies. While this finding is consistent with the notion that cardioembolic strokes often involve larger, more abrupt infarcts with thrombi rich in inflammatory components (18), it is important to note that cardioembolic strokes in our cohort were also associated with greater infarct volumes and higher NIHSS scores. Therefore, the observed differences in chemokine levels may be confounded by stroke severity rather than representing an etiology-specific inflammatory signature. Similarly, the finding of higher IL-8 levels in anterior circulation strokes (particularly MCA territory) may reflect the larger infarct volumes typically seen in these territories rather than a location-specific inflammatory response (8).

Given the unadjusted nature of these analyses and the potential for confounding, these findings should be considered exploratory and hypothesis-generating. Future studies with larger sample sizes and multivariable adjustment for stroke severity, infarct volume, and other clinical factors are needed to determine whether etiological or topographical differences in chemokine levels exist independent of lesion burden.

To facilitate early risk stratification, we constructed a nomogram incorporating five variables available at admission: age, NIHSS score, infarct volume, MCP-1, and IL-8. The nomogram showed good discrimination in the development cohort (C-index 0.872) and maintained acceptable performance in the temporal validation cohort (C-index 0.842). This approach parallels recent work in stroke prognostication; for instance, studies have developed nomograms predicting spontaneous hemorrhagic transformation in non-reperfused AIS patients (9) and pre-hospital delay in AIS patients (10), with AUC values ranging from 0.835 to 0.870.

To rigorously assess the generalizability of our nomogram, we performed a temporal split-sample validation by separating patients into development and validation cohorts based on admission date. The model maintained good discrimination (C-index 0.842) and calibration in the validation cohort, with a net clinical benefit similar to that in the development cohort. These findings suggest that the prognostic value of the nomogram may not be overfitted to the development dataset and could be reproducible in future patients from the same institution. However, external validation in independent, multi-center populations remains essential before widespread clinical adoption.

Although the final nomogram showed good discrimination and calibration, the development cohort contained only 73 poor-outcome events. Even though we restricted the final model to 5 predictors (events-per-variable ratio ≈14.6:1) and performed bootstrap internal validation to correct for optimism, the initial univariate screening and stepwise selection were performed using the same dataset, which may still carry a risk of overfitting or selection bias. Therefore, the model coefficients and performance metrics (e.g., C-index) are likely optimistic. External validation in an independent cohort is essential before the model can be considered generalizable. The reported bootstrap-corrected C-index (0.851) provides a more realistic estimate than the apparent C-index (0.872).

A key methodological consideration of this study is the clear separation between variables available for early risk stratification (admission time) and post-admission events that only become apparent later. While cerebral edema and symptomatic hemorrhage are powerful determinants of functional outcome, they cannot inform decisions made at the time of thrombolysis or initial management. Therefore, our nomogram deliberately excludes these variables, focusing instead on predictors accessible within the first hours of presentation. This design aligns with the clinical objective of early risk stratification. The consistency of age, NIHSS, infarct volume, MCP-1, and IL-8 as independent predictors in both the restricted model and the exploratory model (which included complications) further supports their potential utility as early markers.

Decision curve analysis suggested that the combined model (clinical factors plus biomarkers) provided superior net benefit across threshold probabilities of 10–60% compared with the clinical-only model. At a 30% threshold, the net benefit of 0.28 translates to approximately 9 additional true-positive predictions per 100 patients without increasing false-positives. This finding supports the potential value of incorporating MCP-1 and IL-8 into risk stratification protocols (19), consistent with recent evidence that composite immunoinflammatory indices may predict hemorrhagic transformation in thrombolyzed patients (20).

### Study limitations

Our findings should be interpreted considering several limitations. First, the single-center design and retrospective analysis of clinical data, despite prospective biomarker sampling, necessitate external validation in diverse, multi-center cohorts, including patients undergoing mechanical thrombectomy. While we performed a temporal split-sample validation that supports internal generalizability, this does not replace external validation. Second, the exploratory subgroup analyses examining MCP-1 and IL-8 levels according to TOAST classification and infarct location were unadjusted for potential confounders such as infarct volume and NIHSS score. Although these findings are intriguing and consistent with previous literature, they should be interpreted with caution. The observed differences may be driven by stroke severity rather than etiology or location per se. Adjusted analyses in larger, adequately powered cohorts are necessary to confirm whether these associations are independent. Third, our dynamic analysis was limited to 72 h; longer follow-up could reveal later inflammatory phases relevant to recovery and cognitive outcomes (21). Fourth, while we focused on two key chemokines, the inflammatory network is broader; incorporating markers like IL-6, VCAM-1, or ICAM-1 (11) might enhance model performance. Furthermore, while the final five-predictor model achieved an EPV of 14.6:1, approximately 25 candidate variables were screened during the selection process. The effective EPV at the selection stage was therefore considerably lower, which may have contributed to model instability and optimistic performance estimates. This limitation further underscores the need for external validation. Finally, and most critically, the prognostic model was developed and validated exclusively in AIS patients who received intravenous thrombolysis within 4.5 h of symptom onset. Therefore, the model is not intended for, and should not be applied to, AIS patients treated with mechanical thrombectomy alone, those receiving no reperfusion therapy, or those with delayed presentation beyond the thrombolysis window. The treatment-specific nature of the model is a key scope limitation that must be explicitly acknowledged in any future validation or clinical application. Given the modest number of poor-outcome events in the development cohort, model coefficients should be interpreted with appropriate caution, and the current model should be regarded as requiring further external validation and possible updating in larger datasets.

The results of this study suggest several avenues for future investigation. External validation of the nomogram in independent, multi-center populations is needed before clinical implementation. Interventional studies could explore whether biomarker-guided stratification might improve outcomes by allocating targeted anti-inflammatory therapies to high-risk patients identified by our model (5, 7). Mechanistic studies may help elucidate the cellular and molecular drivers of non-resolving inflammation observed in poor-outcome patients (13, 15).

In summary, this study suggests that in AIS patients receiving intravenous thrombolysis, MCP-1 and IL-8 may serve as dynamic indicators of post-stroke inflammatory trajectory that appear to correlate with recovery potential. The developed nomogram, after further validation, could represent a step toward more personalized prognosis in this treatment-defined population, but external validation in independent cohorts—explicitly restricted to thrombolyzed patients—is essential before clinical application.

## Data Availability

The raw data supporting the conclusions of this article will be made available by the authors, without undue reservation.

## References

[1] Feigin VL Stark BA Johnson CO Roth GA Bisignano C Abady GG . Global, regional, and national burden of stroke and its risk factors, 1990-2019: a systematic analysis for the global burden of disease study 2019. Lancet Neurol. (2021) 20:795–820. doi: 10.1016/S1474-4422(21)00252-0, 34487721 PMC8443449

[2] Goyal M Menon BK van Zwam WH Dippel DW Mitchell PJ Demchuk AM . Endovascular thrombectomy after large-vessel ischaemic stroke: a meta-analysis of individual patient data from five randomised trials. Lancet. (2016) 387:1723–31. doi: 10.1016/S0140-6736(16)00163-X, 26898852

[3] Iadecola C Buckwalter MS Anrather J. Immune responses to stroke: mechanisms, modulation, and therapeutic potential. J Clin Invest. (2020) 130:2777–88. doi: 10.1172/JCI135530, 32391806 PMC7260029

[4] Shi K Tian DC Li ZG Ducruet AF Lawton MT Shi FD. Global brain inflammation in stroke. Lancet Neurol. (2019) 18:1058–66. doi: 10.1016/S1474-4422(19)30078-X, 31296369

[5] Zhu B Liu Y Hwang S . The role of neuroinflammation in ischemic stroke. Front Neurol. (2022) 12:646360. doi: 10.3389/fneur.2021.646360

[6] Georgakis MK Gill D Rannikmäe K Traylor M Anderson CD MEGASTROKE consortium of the International Stroke Genetics Consortium (ISGC) . Genetically determined levels of circulating cytokines and risk of stroke. Circulation. (2019) 139:256–68. doi: 10.1161/CIRCULATIONAHA.118.035905, 30586705 PMC7477819

[7] Ritzel RM Lai YJ Crapser JD Patel AR Schrecengost A Grenier JM . Aging alters the immunological response to ischemic stroke. Acta Neuropathol. (2018) 136:89–110. doi: 10.1007/s00401-018-1859-2, 29752550 PMC6015099

[8] Gülke E Gelderblom M Magnus T. Danger signals in stroke and their role on microglia activation after ischemia. Ther Adv Neurol Disord. (2018) 11:1756286418774254. doi: 10.1177/1756286418774254, 29854002 PMC5968660

[9] Bao J Ma M Wu K Wang J Zhou M Guo J . Integrating neutrophil-to-albumin ratio and triglycerides: a novel Indicator for predicting spontaneous Hemorrhagic transformation in acute ischemic stroke patients. CNS Neurosci Ther. (2024) 30:e70133. doi: 10.1111/cns.70133, 39690502 PMC11652394

[10] Damon MS Basse AM Sow AD Bassole PR Diop-Sene MS Banzouzi FL . Pre-hospital delay in patients with ischemic stroke in the Fann teaching hospital, Dakar, Senegal in 2020. Pan Afr Med J. (2022) 41:79. doi: 10.11604/pamj.2022.41.79.30191, 35382054 PMC8956836

[11] Bustamante A López-Cancio E Pich S Penalba A Giralt D García-Berrocoso T . Blood biomarkers for the early diagnosis of stroke: the stroke-Chip study. Stroke. (2017) 48:2419–25. doi: 10.1161/STROKEAHA.117.017076, 28716979

[12] Xu Q Liu Y Tian X Xia X Zhang Y Zhang X . Monocyte chemoattractant protein-1, inflammatory biomarkers, and prognosis of patients with ischemic stroke or transient ischemic attack: fundings from a nationwide registry study. J Am Heart Assoc. (2024) 13:e035820. doi: 10.1161/JAHA.124.035820, 39119971 PMC11963953

[13] Jayaraj RL Azimullah S Beiram R Jalal FY Rosenberg GA. Neuroinflammation: friend and foe for ischemic stroke. J Neuroinflammation. (2019) 16:142. doi: 10.1186/s12974-019-1516-2, 31291966 PMC6617684

[14] Jickling GC Liu D Ander BP Stamova B Zhan X Sharp FR. Targeting neutrophils in ischemic stroke: translational insights from experimental studies. J Cereb Blood Flow Metab. (2015) 35:888–901. doi: 10.1038/jcbfm.2015.45, 25806703 PMC4640255

[15] Ma Y Wang J Wang Y Yang GY. The biphasic function of microglia in ischemic stroke. Prog Neurobiol. (2017) 157:247–72. doi: 10.1016/j.pneurobio.2016.01.005, 26851161

[16] Rapido F Marchi N Labreuche J ter Schiphorst A Blaquière M de Bock F . Acute ischemic stroke and reperfusion drive molecular immune-vascular activations detectable in peripheral blood. J Neurointerv Surg. (2025). doi: 10.1136/jnis-2025-023885, 40987590

[17] Campbell RA Vieira-de-Abreu A Rowley JW Franks ZG Manne BK Rondina MT . Clots are potent triggers of inflammatory cell gene expression: indications for timely fibrinolysis. Arterioscler Thromb Vasc Biol. (2017) 37:1819–27. doi: 10.1161/ATVBAHA.117.309794, 28775073 PMC5620127

[18] Kamtchum-Tatuene J Jickling GC. Blood biomarkers for stroke differentiation. Curr Neurol Neurosci Rep. (2020) 20:21. doi: 10.1007/s11910-020-01038-2, 32444979

[19] Van Calster B Wynants L Verbeek JFM Verbakel JY Christodoulou E Vickers AJ . Reporting and interpreting decision curve analysis: a guide for investigators. Eur Urol. (2018) 74:796–804. doi: 10.1016/j.eururo.2018.08.038, 30241973 PMC6261531

[20] Cai J Rao H Li X Luo J Wang Z Liu D. Predictive value of the modified comprehensive immunoinflammatory indices for hemorrhagic transformation in ischemic stroke patients undergoing thrombolysis: a retrospective study. Int J Gen Med. (2025) 18:6353–63. doi: 10.2147/IJGM.S545665 41141888 PMC12553340

[21] Montellano FA Ungethüm K Ramiro L Nacu A Hellwig S Fluri F . Role of blood-based biomarkers in ischemic stroke prognosis: a systematic review. Stroke. (2021) 52:543–51. doi: 10.1161/STROKEAHA.120.029232, 33430636

