# Supplementary material for: A prognostic model incorporating MCP-1 and IL-8 for early risk stratification in acute ischemic stroke patients receiving intravenous thrombolysis: a retrospective development with temporal validation
Source: Front Neurol. 2026 Jun 4;17:1765374. doi: 10.3389/fneur.2026.1765374 (PMC13275240; doi:10.3389/fneur.2026.1765374)
Supplement: Supplementary file 1 [file Table_1.docx]

**Table S1. Multivariable Linear Regression Analysis of Factors Associated with MCP-1 and IL-8 Levels**

| **Variable** | **MCP-1 (β, 95% CI)** | **P** | **IL-8 (β, 95% CI)** | **P** |
| --- | --- | --- | --- | --- |
| TOAST (ref: atherosclerosis) |  |  |  |  |
| Cardioembolism | 18.5 (-2.3, 39.3) | 0.081 | 5.2 (-0.8, 11.2) | 0.089 |
| Other etiology | -8.2 (-28.6, 12.2) | 0.432 | -3.1 (-9.5, 3.3) | 0.342 |
| Infarct location (ref: posterior) |  |  |  |  |
| Anterior circulation | 5.1 (-12.4, 22.6) | 0.569 | 6.8 (1.2, 12.4) | 0.018 |

*Note: Models adjusted for age, NIHSS score, infarct volume, hypertension, diabetes, and atrial fibrillation.*

Table S2 **Baseline Characteristics of Development and Validation Cohorts**

| Characteristic | Development Cohort (n=200) | Validation Cohort (n=100) | P Value |
| --- | --- | --- | --- |
| Demographics |  |  |  |
| Age (years), mean ± SD | 64.5 ± 11.2 | 65.1 ± 10.9 | 0.654 |
| Male, n (%) | 118 (59.0) | 64 (64.0) | 0.402 |
| Vascular risk factors, n (%) |  |  |  |
| Hypertension | 108 (54.0) | 62 (62.0) | 0.184 |
| Diabetes mellitus | 52 (26.0) | 33 (33.0) | 0.197 |
| Atrial fibrillation | 35 (17.5) | 23 (23.0) | 0.253 |
| Previous stroke/TIA | 27 (13.5) | 16 (16.0) | 0.556 |
| Clinical and imaging parameters |  |  |  |
| Admission NIHSS score, median (IQR) | 9.0 (6.0–13.0) | 9.0 (6.0–14.0) | 0.613 |
| Infarct volume (mL), median (IQR) | 24.3 (18.5–30.2) | 25.0 (19.0–31.5) | 0.482 |
| OTT (h), mean ± SD | 2.6 ± 1.0 | 2.7 ± 1.1 | 0.421 |
| Inflammatory biomarkers |  |  |  |
| MCP-1 (pg/mL), median (IQR) | 215.4 (180.2–250.6) | 213.8 (178.5–249.1) | 0.817 |
| IL-8 (pg/mL), median (IQR) | 79.6 (68.3–90.9) | 78.9 (67.5–90.3) | 0.762 |
| Outcome |  |  |  |
| Poor outcome (mRS>2), n (%) | 73 (36.5) | 35 (35.0) | 0.793 |

**Table S3. Exploratory Multivariate Analysis Including Post-Admission Complications**

| Variable | β | SE | Wald χ² | P | OR | 95% CI |
| --- | --- | --- | --- | --- | --- | --- |
| Age | 0.047 | 0.012 | 15.3 | <0.001 | 1.048 | 1.024–1.073 |
| Admission NIHSS score | 0.219 | 0.048 | 20.83 | <0.001 | 1.245 | 1.133–1.368 |
| Infarct volume | 0.081 | 0.021 | 14.96 | <0.001 | 1.084 | 1.041–1.129 |
| MCP-1 | 0.012 | 0.004 | 9.22 | 0.002 | 1.012 | 1.004–1.021 |
| IL-8 | 0.025 | 0.008 | 9.77 | 0.002 | 1.025 | 1.009–1.041 |
| Cerebral edema | 0.924 | 0.332 | 7.73 | 0.005 | 2.519 | 1.323–4.793 |
| Symptomatic hemorrhage | 1.136 | 0.441 | 6.64 | 0.01 | 3.115 | 1.316–7.377 |
